# [Retracted] Actin‑binding protein anillin promotes the progression of hepatocellular carcinoma in vitro and in mice

**DOI:** 10.3892/etm.2023.12120

**Published:** 2023-07-17

**Authors:** Huanxia Jia, Zhenya Gao, Fang Yu, Hongfang Guo, Baoyu Li

Exp Ther Med 21:454, 2021; DOI: 10.3892/etm.2021.9885

Following the publication of the above paper, it was drawn to the Editor’s attention by a concerned reader that certain of the migration and invasion assay data shown in Fig. 4D (in addition to an internal duplication of data within Fig. 4D itself) and tumour images shown in Fig. 5A, all on p. 7, were strikingly similar to data that had already appeared in previous publications (one of which has been retracted).

Owing to the fact that the contentious data in the above article had already been published elsewhere, or were under consideration for publication, prior to its submission to *Experimental and Therapeutic Medicine*, the Editor has decided that this paper should be retracted from the Journal. The authors were asked for an explanation to account for these concerns, but the Editorial Office did not receive a reply. The Editor apologizes to the readership for any inconvenience caused.

